# The Effects of the Natriuretic Peptide System on Alveolar Epithelium in Heart Failure

**DOI:** 10.3390/ijms26073374

**Published:** 2025-04-04

**Authors:** Yara Knany, Safa Kinaneh, Emad E. Khoury, Yaniv Zohar, Zaid Abassi, Zaher S. Azzam

**Affiliations:** 1 Department of Physiology and Biophysics, The Ruth and Bruce Rappaport Faculty of Medicine, Technion, Israel Institute of Technology, Haifa 3109601, Israel; yara.knany@mail.huji.ac.il (Y.K.); safakinaneh@gmail.com (S.K.); emadkhoury92@gmail.com (E.E.K.); y_zohar@rambam.health.gov.il (Y.Z.); abassi@technion.ac.il (Z.A.); 2 Department of Otolaryngology-Head and Neck Surgery, Rambam Health Care Campus, Haifa 3109601, Israel; 3 Institute of Pathology, Rambam Health Care Campus, Haifa 3109601, Israel; 4 Department of Medicine, Management, Rambam Health Care Campus, Haifa 3109601, Israel

**Keywords:** natriuretic peptides, alveolar fluid clearance, heart failure, active sodium transport, ubiquitination

## Abstract

Alveolar active sodium transport is essential for clearing edema from airspaces, in a process known as alveolar fluid clearance (AFC). Although it has been reported that atrial natriuretic peptide (ANP) attenuates AFC, little is known about the underlying molecular effects of natriuretic peptides (NPs). Therefore, we examined the contribution of NPs to AFC and their effects as mediators of active sodium transport. By using the isolated liquid-filled lungs model, we investigated the effects of NPs on AFC. The expression of NPs, Na^+^, K^+^-ATPase, and Na^+^ channels was assessed in alveolar epithelial cells. Congestive heart failure (CHF) was induced by using the aortocaval fistula model. ANP and brain NP (BNP) significantly reduced AFC rate from 0.49 ± 0.02 mL/h in sham rats to 0.26 ± 0.013 and 0.19 ± 0.005 in ANP and BNP-treated groups, respectively. These effects were mediated by downregulating the active Na^+^ transport components in the alveolar epithelium while enhancing the ubiquitination and degradation of αENaC in the lungs, as reflected by increased levels of Nedd4-2. In addition, AFC was reduced in compensated CHF rats treated with ANP, while in decompensated CHF, ANP partially restored AFC. In conclusion, NPs regulate AFC in health and CHF. This research could help optimize pharmacological treatments for severe CHF.

## 1. Introduction

Cardiogenic pulmonary edema is a severe form of decompensated congestive heart failure (CHF). In recent decades, despite improvements in treatment, CHF remains a global health problem with excess morbidity and mortality, particularly amongst the elderly [1]. The pathophysiology of heart failure is characterized by hyper-activation of the sympathetic nervous system, reninan–giotensin–aldosterone system, and vasopressin [2,3,4] with resultant aggravation of CHF. Conversely, natriuretic peptides (NPs) exert a compensatory action on CHF. Notably, there are three known NPs: atrial natriuretic peptide (ANP, previously called atrial natriuretic factor—ANF), brain natriuretic peptide (BNP), and C-type natriuretic peptide (CNP). ANP and BNP have beneficial effects on CHF by causing vasodilatation, inhibiting the secretion of renin and aldosterone, and antagonizing the sympathetic system [5,6,7]. However, one of the puzzling issues in the pathogenesis of heart failure is the dominance of Na^+^ retaining/neurohumoral vasoconstrictor systems over NPs.

Active alveolar fluid clearance (AFC) plays a crucial role in maintaining a free airspace from edema. This process is mediated through active sodium transport, where Na^+^ is extruded from the alveolar airspace by epithelial transport proteins, including apical epithelial Na^+^ channels (ENaC) and basolateral Na^+^, K^+^-ATPases [8]. The alveolar epithelium, especially alveolar epithelial cell type II (AECII), is actively involved in the pathogenesis of heart failure. AECII constitute 15% of distal lung cells and cover 4% of the lung’s surface. These small, cuboidal cells store surfactant in lamellar bodies and are responsible for synthesizing, secreting, and reabsorbing pulmonary surfactant. AECII also play a role in AFC by actively transporting sodium. As progenitor cells, they proliferate and differentiate into AECI cells following injury. Surfactant protein C (SP-C), specific to AECII, is used for identification, and its expression decreases when AECII differentiate into AECI-like cells [9]. By investigating the role of alveolar epithelial active sodium transport and alveolar fluid clearance (AFC), others and we found an acute increase in left atrial pressure that led to a decline in AFC [10,11,12]. On the other hand, chronic elevation of pulmonary capillary pressure, such as seen in experimental and clinical CHF, caused an increase in the rate of edema clearance by upgrading the active transport of sodium [13]. Olivera et al. examined the effect of ANP on AFC and reported that ANP impeded AFC by increasing alveolar epithelial permeability and decreasing active Na^+^ transport out of the airspaces. Notably, the levels of c-GMP were increased in response to ANF administration; therefore, they presumed that these changes possibly induced blockage of apical Na^+^ channels [14]. This study provides physiologic evidence of the ANF effect on active Na^+^ transport and AFC. Recently, Khoury et al. demonstrated through Western blot analysis that ANP and BNP were present in pulmonary tissue where their levels were elevated in the lungs of both compensated and decompensated CHF rats [15]. There are three identified natriuretic peptide receptors: guanylyl cyclase-A/natriuretic peptide receptor-A (GC-A/NPR-A), GC-B/NPR-B, and NPR-C. NPR-A and NPR-B are primarily responsible for mediating the full range of biological effects through the guanylate cyclase (GC) intracellular signaling pathway, whereas NPR-C is not associated with GC activity and functions instead as a clearance receptor [7].

Given the adverse effect of ANP on lung edema clearance, we hypothesized that NPs may have a role in the process of alveolar active sodium transport and AFC, both in normal condition and edematous disease states such as CHF. Therefore, the aims of this trial are to investigate (1) NPs system involvement in AFC in both normal conditions and an experimental model of heart failure; (2) the effects of ANP on AFC-related proteins, including Na^+^, K^+^-ATPase, and ENaC in lung tissues; and (3) the mechanisms by which NPs modulate active sodium transport components.

## 2. Results

### 2.1. Stage 1: Effect of Natriuretic Peptides on AFC in Normal Lung

**Figure 1:**
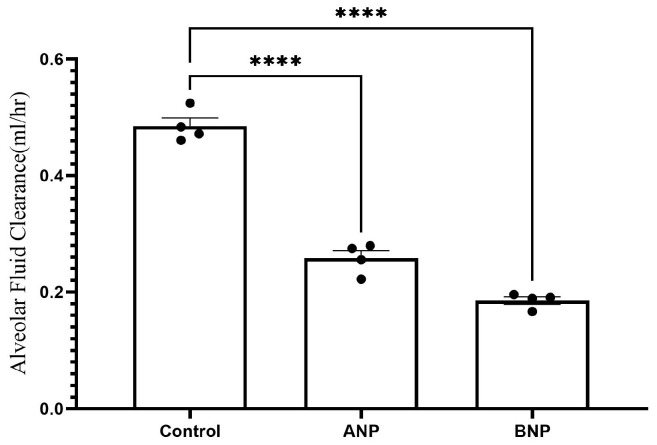
Effect of NPs on AFC. The rate of alveolar fluid clearance was decreased in the ANP- and BNP-treated groups, from 0.485 ± 0.01 mL/h in control rats (*n* = 4) to 0.2581 ± 0.013 mL/h (*n* = 4) and 0.1856 ± 0.005 mL/h (*n* = 4) in ANP- and BNP-treated groups, respectively. **** *p* < 0.001 represents a significant difference in the NP groups as compared to controls. (one-way ANOVA Kruskal–Wallis test). Bars represent mean ± SEM.

The rate of AFC was significantly decreased in ANP-treated lungs as compared with the control group) 0.485 ± 0.01 mL/h vs. 0.2581 ± 0.01302 mL/h, respectively, *p* < 0.0001). Similarly, the AFC rate in BNP-treated lungs significantly declined as compared to the control group (0.1856 ± 0.005 mL/h vs. 0.485 ± 0.01 mL/h, respectively, *p* < 0.0001) as presented in Figure 1.

### 2.2. In Vivo Protocols: Normal Rats

Since we found that both ANP and BNP impeded AFC, we decided to use ANP as a representative of NPs and determine the mechanisms by which AFC was impeded. We investigated the effects of ANP on the following target proteins: NPR-A, Na^+^, K^+^-ATPase pump, and α-ENaC in whole lung tissue and AECII. The following target proteins were examined at both mRNA and protein levels at 24 h following the administration of ANP.

#### 2.2.1. ANP and BNP Effect on NPR-A Expression in Lung Tissue

To determine whether the adverse effect of ANP and BNP on AFC is mediated by alterations in the pulmonary ANP, BNP receptor. NPR-A mRNA levels were measured 24 h following IV injection of ANP/BNP/saline. By using the RT-PCR methodology, the amounts of NPR-A in the lungs of all experimental groups were not different (Figure S2). These results indicate that the ANP and BNP effect on AFC is not mediated by the regulation of the NPR A expression in lung tissue.

#### 2.2.2. ENaC

**Figure 2:**
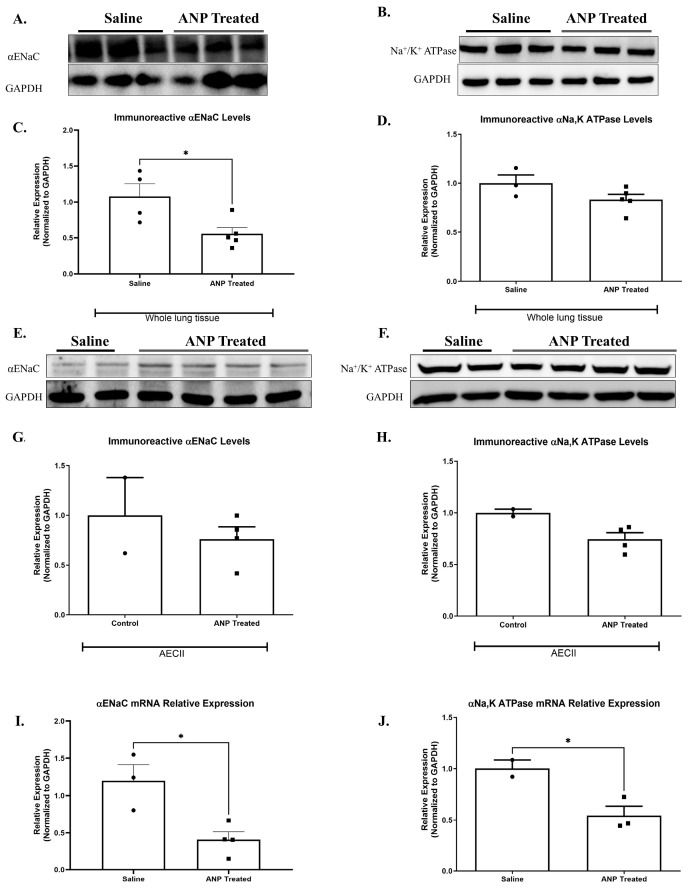
Effect of ANP treatment on αENaC and Na^+^, K^+^ATPase levels. Representative Western blot analysis of αENaC and Na^+^, K^+^ ATPase in whole lung tissue lysate and isolated AECII are shown in (**A**,**B**,**E**,**F**), respectively. (**C**) Western blot analysis relative quantification of αENaC in lung tissue (saline-treated group *n* = 4. ANP-treated group *n* = 5). (**D**) Western blot analysis relative quantification of Na^+^, K^+^-ATPase in lung tissue (saline-treated group *n* = 3. ANP-treated group *n* = 5). (**G**,**H**) Representative Western blot analysis relative quantification of αENaC and Na^+^, K^+^-ATPase in isolated AECII (normal group *n* = 2. ANP-treated group *n* = 4). GAPDH was used as a loading control. Quantification of qPCR analysis of αENaC and Na^+^, K^+^-ATPase mRNA in isolated AECII, normalized to GAPDH, is depicted in (**I**,**J**), respectively. * *p* < 0.05 represents significant differences between saline-treated groups vs. ANP-treated groups. Values are means ± SEM. (*t*-test).

There was a significant decrease in the abundance of α-ENaC in whole lung tissue in Western blot analysis (Figure 2A,C). However, the reduction of ENaC in isolated AECII was not statistically significant (Figure 2E,G). By using qPCR analysis, α-ENaC mRNA expression in isolated AECII was found to remarkably decrease following ANP treatment, as compared to the control group (*p* < 0.05) (Figure 2I).

#### 2.2.3. Na^+^, K^+^-ATPase

Since NPs impeded AFC, we investigated the effect of ANP on α1-Na^+^, K^+^-ATPase protein by examining abundance in both whole cell tissue and isolated AECII. We found a slight decrease, not statistically significant, in ANP-treated groups in both whole lung tissue (Figure 2B,D) and AECII (Figure 2F,H). By using qPCR analysis, α1-Na^+^, K^+^-ATPase mRNA expression in isolated AECII were found to remarkably decrease following ANP treatment, as compared to the control group (Figure 2J).

**Figure 3:**
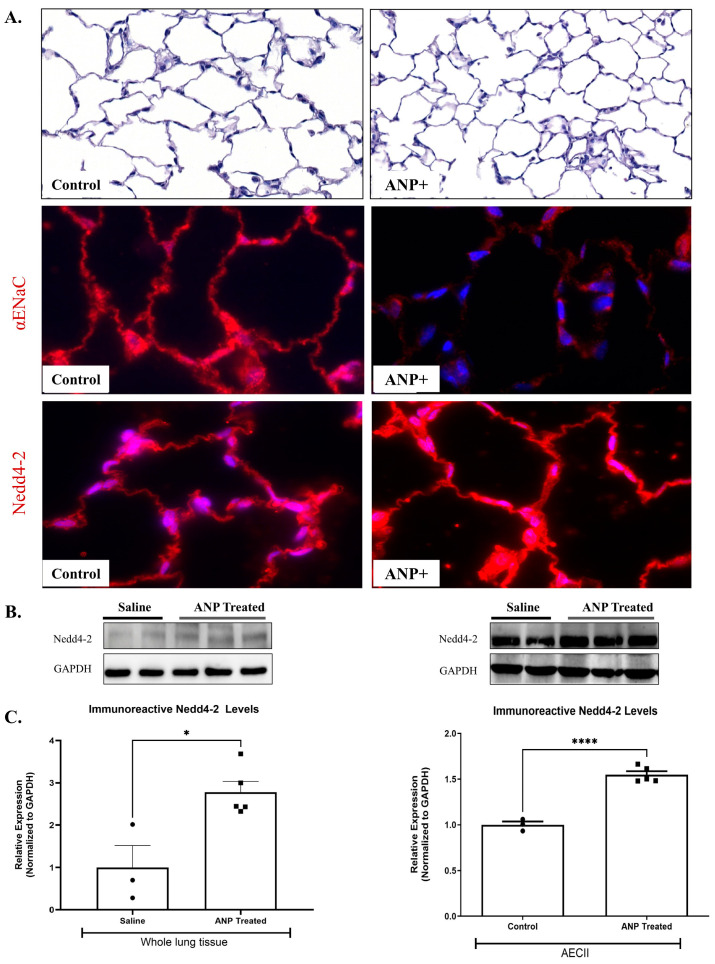
Effect of ANP treatment on αENaC ubiquitination. αENaC and Nedd4-2 immunofluorescence staining in lung tissue of ANP-treated rats and their control that were treated with saline. (**A**) Immunofluorescence detected by CY3-conjugated secondary antibodies (shown in red), magnification ×20 scale bar 10 µm. Representative Western blot analysis Nedd4-2 in whole lung tissue lysate and isolated AECII are shown in (**B**). (**C**) Western blot analysis relative quantification of Nedd4-2 in lung tissue and isolated AECII (saline-treated group *n* = 3, ANP-treated group *n* = 5). GAPDH was used as a loading control. * *p* < 0.05 and **** *p* < 0.001 represent significant differences between saline-treated groups vs. ANP-treated groups. (*t*-test). Values are means ± SEM.

Immunofluorescent staining against α-ENaC in the control lungs demonstrated that ANP-treated rats resulted in α-ENaC downregulation (Figure 3A).

#### 2.2.4. α-ENaC Ubiquitination

To identify the mechanisms by which ANP downregulates α ENaC, we assessed the effects of ANP on Nedd4-2, an E3 ubiquitin-protein ligase that is important for ENaC regulation. By using Western blot analysis, we detected a significant increase in Nedd4-2 protein levels in both whole lung tissues and AECII of ANP-treated groups (Figure 3B,C). These findings were further supported by the immunofluorescence staining against Nedd4-2 in the lung tissue (Figure 3A). Therefore, it is conceivable to presume that these findings support the hypothesis that the adverse effects of ANP on AFC were exerted via ubiquitination of the apical α-ENaC in AEC.

### 2.3. Electron Microscopy

**Figure 4:**
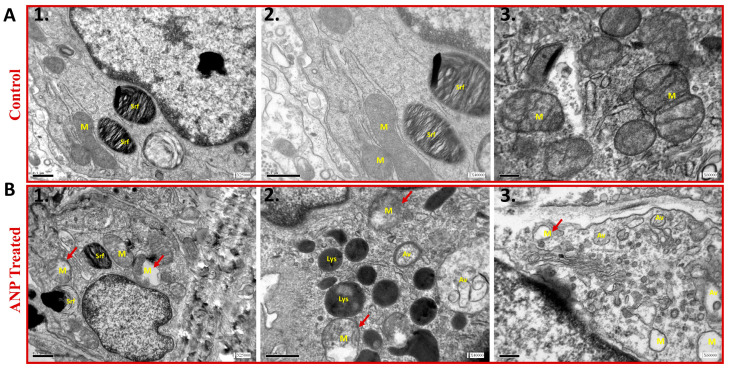
The effect of ANP on AECII ultrastructure. Transmission electron microscopy images of AECII of ANP-treated rats (Panel **B**) and their controls that were treated with saline (Panel **A**). The images in Panel **A1**–**3** demonstrate normal morphology of AECII; whereas ANP treatment induced abnormal morphology with mitochondrial destruction and damage (red arrowhead) with matrix swelling and collapsed cristae (**B1**,**2**,**3**). In addition, autophagic vacuoles were observed in ANP-treated cells (**B2**,**3**). Lysosomal accumulation was also observed in the treated group (**B2**). M–mitochondria, Lys–lysosome, Srf–surfactant, Av–autophagic vacuoles. Scale bar **A1**–**2**, **B1**–**2**: 0.5 μm. Scale bar **A3**, **B3**: 0.2 μm.

To further examine the involvement of ANP in alveolar epithelial function, lung ultrastructural morphology was evaluated in both sham ANP-treated rats and their controls by using electron microscopy analysis. As compared to control (Figure 4A), the mitochondria of ANP-treated cells were damaged and destroyed with disorganization of the cristae (Figure 4B). We also observed that in ANP-treated groups, AECs seemed to have an increased number of lysosomes and cytoplasmic vacuoles suggestive of typical autophagosomes. Notably, these vacuoles were fused into larger vacuoles as compared to the untreated control group. Therefore, it is conceivable to conclude that treatment with ANP induced autophagy.

### 2.4. Stage 2: Lung Tissue of Congestive Heart Failure Rats

#### 2.4.1. Effect of ANP on AFC in CHF

**Figure 5:**
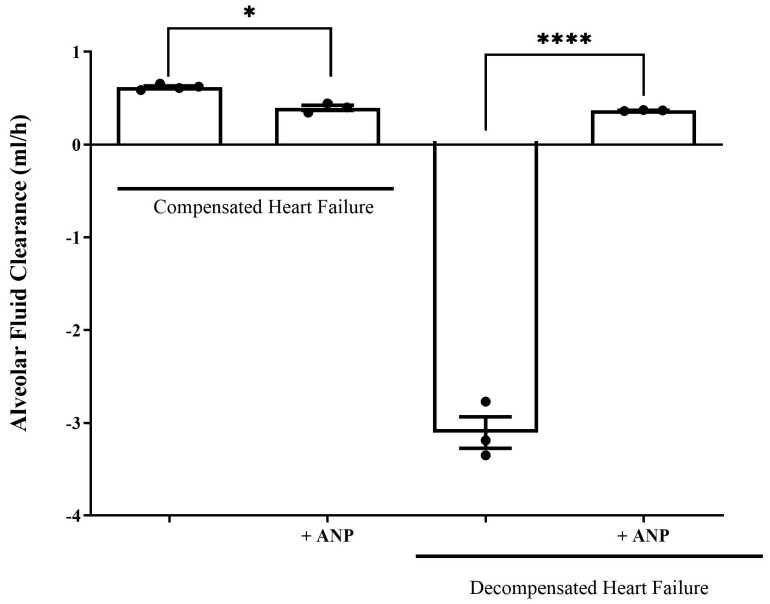
Effect of NPs on AFC rate in both compensated and decompensated rats. The rate of alveolar fluid clearance was significantly decreased in compensated CHF rats treated with ANP (0.3967 ± 0.03180 mL/h, *n* = 3) as compared to the untreated compensated CHF group (0.621 ± 0.014 mL/h (*n* = 4) * *p* = 0.033). In decompensated CHF rats, AFC was drastically declined; however, the administration of ANP restored AFC from −3.107 ± 0.1821 mL/h (*n* = 3) to 0.3667 ± 0.00393 mL/h (*n* = 3). **** *p* = 0.0495. Results are expressed as mean ± SEM (Wilcoxon–Mann–Whitney test).

The ability of the lungs to clear edema was significantly decreased in compensated HF rats that were treated with ANP as compared to untreated compensated HF lungs (0.621 ± 0.014 mL/h vs. 0.3967 ± 0.03180 mL/h, respectively, *p* = 0.0331). In contrast, treatment with ANP significantly restored the rate of AFC in decompensated CHF rats from −3.107 ± 0.1821 mL/h to 0.3677 ± 0.0039 mL/h (*p* = 0.0495) (Figure 5). Notably, the lungs of the decompensated rats were larger and more edematous.

#### 2.4.2. Immunofluorescence

**Figure 6:**
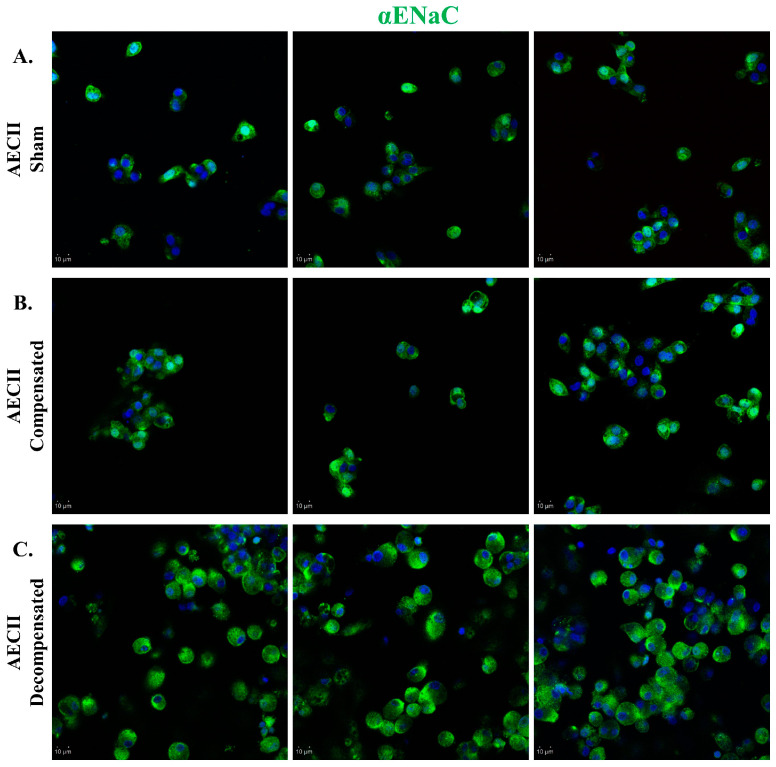
αENaC expression in CHF. Immunofluorescence analysis of αENaC in AECII isolated from sham control (**A**), compensated CHF (**B**), and decompensated CHF (**C**). Cells were stained with antibodies against αENaC, and immunofluorescence was detected by Alexa fluor 488-conjugated secondary antibodies (shown in green). In decompensated HF rats, there was a remarkable upregulation of ENaC as compared to sham-operated rats. Images were obtained by a confocal LSM 880 upright microscope. Magnification ×20. Scale bar = 10 μm.

Immunofluorescent staining of AEC against α-ENaC in compensated HF demonstrated a slight upregulation of ENaC in comparison to sham-operated rats. The levels of ENaC in the AEC cells isolated from the decompensated HF rats appeared to be increased compared to the levels seen in the sham group (Figure 6).

#### 2.4.3. The Effect of ANP on the Mediators of Alveolar Active Sodium Transport in CHF

**Figure 7:**
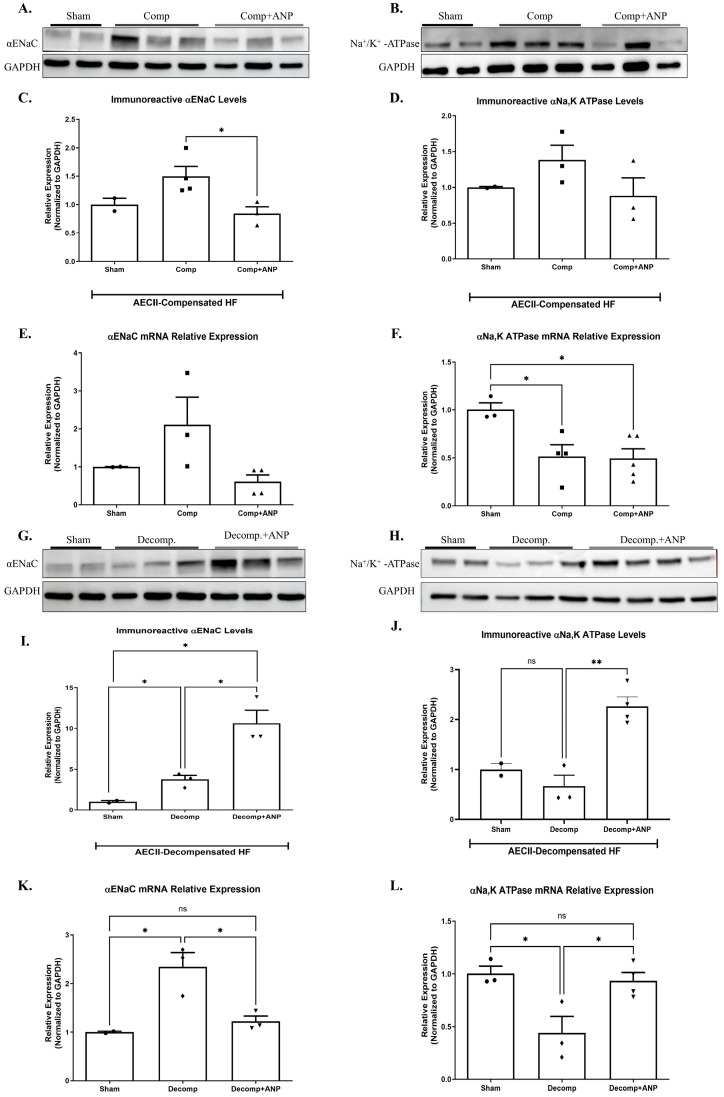
The effect of ANP treatment on levels and expressions of αENaC and Na^+^, K^+^-ATPase in compensated CHF and decompensated CHF. Representative Western blot analysis of αENaC and Na^+^, K^+^-ATPase in isolated AECII are shown in (**A**,**B**,**G**,**H**), respectively. (**C**) Western blot analysis relative quantification of αENaC in isolated AECII (sham-*n* = 2, compensated-*n* = 4, compensated treated with ANP–*n* = 3). (**D**) Western blot analysis relative quantification of Na^+^, K^+^ ATPase in isolated AECII (sham-*n* = 2, compensated-*n* = 3, compensated treated with ANP–*n* = 3). Quantification of qPCR analysis of αENaC mRNA in isolated AECII from compensated CHF and compensated CHF-ANP-treated group (**E**) and sham-*n* = 2, compensated-*n* = 3, and compensated treated with ANP–*n* = 4) (**F**) Na^+^, K^+^-ATPase mRNA in isolated AECII from compensated CHF and compensated CHF-ANP treated group (sham-*n* = 3, compensated-*n* = 4, compensated treated with ANP–*n* = 5). (**I**) Western blot analysis relative quantification of αENaC ATPase isolated AECII from the decompensated groups and the decompensated ANP-treated group (sham-*n* = 2, decompensated-*n* = 3, decompensated treated with ANP–*n* = 3). (**J**) relative quantification of Na^+^, K^+^ ATPase levels in ATPase-isolated AECII from the decompensated groups and the decompensated ANP-treated group (sham-*n* = 2, decompensated-*n* = 3, decompensated treated with ANP–*n* = 4). Quantification of qPCR analysis of αENaC mRNA levels in AECII isolated from decompensated and decompensated—ANP-treated rats is depicted in (**K**) (sham-*n* = 2, decompensated-*n* = 3, decompensated treated with ANP–*n* = 3). (**L**) mRNA levels quantification of Na^+^, K^+^-ATPase in AECII isolated from decompensated and decompensated—ANP-treated rats are depicted in (**K**) (sham-*n* = 3, decompensated-*n* = 3, decompensated treated with ANP–*n* = 4). All results were normalized to GAPDH levels. * *p* < 0.05, ** *p* < 0.01. The bars represent mean ± SEM (Wilcoxon–Mann–Whitney test). ns: nonsignificant.

To further investigate the involvement of ANP in the pathophysiology of the lungs in CHF, we examined the effect of ANP treatment on the expression of α-ENaC and Na^+^, K^+^-ATPase in isolated AECII of both compensated and decompensated HF rats. α-ENaC immunoreactivity of ANP treated compensated CHF rats in AECII was found to be significantly decreased as compared to sham-operated rats and untreated compensated rats (Figure 7A,C). Notably, the expression of α-ENaC mRNA in isolated AECII from compensated CHF rats was also decreased following ANP treatment, as compared to control and untreated compensated HF; however, it was not significant (Figure 7E).

As depicted in Figure 7B,D, Na^+^, K^+^-ATPase tended to be decreased in compensated HF rats treated with ANP as in other study groups. The expression of Na^+^, K^+^-ATPase mRNA was significantly decreased following the administration of ANP as compared to sham-operated rats (Figure 7F).

The mediators of alveolar epithelial active transport were investigated in AECII of rats with decompensated CHF that had received ANP. Figure 7G,I showed a significant upregulation in α-ENaC subunit in both decompensated groups as compared to sham-operated rats. It is worth mentioning that the increase was more prominent in the ANP-treated group. α-ENaC mRNA expression was also augmented in decompensated HF with partial attenuation in the ANP-treated group (Figure 7K).

Na^+^, K^+^-ATPase immunoreactivity and m-RNA were both downregulated in AECII of decompensated HF; however, in comparison with the decompensated group, ANP significantly upregulated Na^+^, K^+^-ATPase (Figure 7J,L).

### 2.5. Stage 3: ANP Regulates αENaC Levels via Nedd4-2

To investigate the interplay of ANP with αENaC levels, we sought to determine whether the effect of ANP on αENaC was mediated via ENaC ubiquitination and whether this correlated with changes in Nedd4-2 levels.

**Figure 8:**
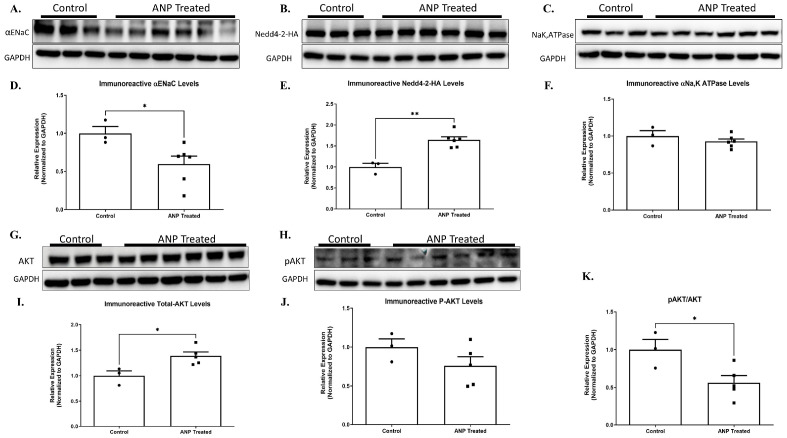
ANP-induced αENaC ubiquitination. Representative Western blot analysis of αENaC, Nedd4-2-HAtag, and Na^+^, K^+^-ATPase HEK cells that were co-transfected with Nedd4-2-HA tag and PGC plasmids are shown in (**A**–**C**), respectively. (**D**–**F**) Western blot analysis relative quantification of αENaC, Nedd4-2-HAtag, and Na+, K+-ATPase HEK co-transfected cells after treatment with ANP. GAPDH was used as a loading control. (**G**,**H**) Representative Western blot analysis of AKT and p-AKT in co-transfected cells after ANP treatment, respectively. (**I**,**J**) Western blot analysis relative quantification of AKT and p-AKT in co-transfected cells treated with ANP. (**K**) displays pAKT/AKT ratio in co-transfected cells that were treated with ANP as compared to their controls. Control group *n* = 3, ANP-treated group *n* = 6. * *p* < 0.05, ** *p* < 0.001 represent significant differences between the control group and the ANP-treated group. Values are means ± SEM (*t*-test).

Our results showed that for Nedd4-2 co-transfected cells that were treated with ANP in pGC-A, αENaC levels were significantly downregulated as compared to co-transfected cells that had not been treated with ANP (Figure 8A,D). Na^+^, K^+^-ATPase was not affected by ANP treatment (Figure 8C,F).

αENaC downregulation seemed to be related to Nedd4-2 regulation. Our results showed that ANP treatment triggered Nedd4-2 upregulation. As depicted in Figure 8B,E Western blot results determined that the co-transfected cells significantly increased Nedd4-2 levels following ANP treatment as compared to co-transfected cells of the controls. These results indicate that ANP downregulates αENaC via Nedd4-2, which leads to αENaC ubiquitination and removal from the membrane.

To further investigate the molecular pathway by which ANP affects α ENaC ubiquitination, we investigated the effect of ANP on Nedd4–2 function. According to recent reports, certain kinases, namely Sgk1 and Akt, facilitate the phosphorylation of Nedd4-2 at specific serine residues. This, in turn, enhances the recruitment and binding of 14-3-3 proteins 2 [16,17,18]. The function of Nedd4-2 is controlled by the binding of 14-3-3 proteins, which act as regulators by inhibiting the interaction between Nedd4-2 and its substrates, specifically αENaC. This interaction is important for the proper functioning of Nedd4-2, and the inhibition of this interaction by 14-3-3 proteins can have significant implications in various biological processes [18]. For this reason, we tested whether AKT and phosphorylated AKT (p-AKT) levels were changed in the ANP-treated cells. The findings of our study reveal a significant increase in AKT levels in co-transfected cells subjected to ANP treatment as compared to the control group. However, it is noteworthy that we did not observe a concomitant increase in the phosphorylation of AKT levels upon ANP treatment, as evidenced by Figure 8I,J.

These changes correlated with the upregulation of the nedd4-2, with a 2-fold decrease in αENaC levels because of ANP treatment. The increase of AKT levels along with the remarkable decrease in pAKT/AKT ratio (Figure 8I,J,K) provides evidence that ANP has a role in mediating Nedd4-2 phosphorylation, which may lead to improved Nedd4-2 function that could result in αENaC hyper-ubiquitination along with consequent impairment of Na^+^ transport in the lung.

## 3. Discussion

The capability of the lungs to clear edema in health and in heart failure can be modulated by several factors such as catecholamines, vasopressin, angiotensin, and ANP, previously known as ANF [19].

In agreement with a previous observation that ANP impeded AFC by blocking alveolar epithelial sodium channels and increasing lung epithelial permeability possibly via c-GMP [14], we found that both ANP and BNP decreased AFC rate by more than 50% in sham rats (Figure 1). However, in our study, we concentrated on investigating the molecular mechanisms of NPs effects.

To understand the underlying molecular mechanisms of the inhibitory effects of NPs on AFC, we sought to examine the effects of ANP on the main components of alveolar active sodium transport. We found that the expression of α-ENaC and Na^+^, K^+^-ATPase m-RNA in ANP-treated AECII remarkably declined. In agreement with this finding, the immunoreactivity of these parameters was also decreased, yet to a lesser extent. Notably, α-ENaC was significantly decreased in whole lung tissue treated with ANP (Figure 2). In agreement with these observations, several studies have reported that ANP produces natriuretic and diuretic effects in the kidney by inhibiting both the basolaterally expressed Na^+^, K^+^ ATPase pump and apical α-ENaC [20,21,22].

There is growing evidence that Nedd4-2 downregulates ENaC expression in the apical membrane of epithelial cells, leading to a significant reduction in its levels [23,24,25]. Additionally, in total lung extracts in newborn rats, Nedd4-2 silencing increased the expression of α-ENaC and β-ENaC protein levels with a consequent decrease of extravascular lung waters [26]. Concordantly, we have shown, in both whole lung tissue and AECII, that ANP treatment enhanced the ubiquitination and the degradation of α-ENaC by upregulating the Nedd4-2 levels (Figure 3). Therefore, it is conceivable to suggest that Nedd4-2 enhanced alveolar α-ENaC degradation with resultant attenuation of alveolar epithelial active Na^+^ transport components and AFC. Notably, although the involvement of Nedd4-2 in epithelial Na^+^ transport was studied in various tissues and organs, to the best of our knowledge, this study is the first to examine the effect of ANP on α ENaC ubiquitination in lung tissue. Therefore, these sets of experiments indicate that ANP decreased AFC by downregulating the α1 Na^+^, K^+^-ATPase pump and enhancing the α ENaC ubiquitination and degradation, thus contributing to pulmonary congestion.

Further insights into the lung ultrastructural morphology were obtained by electron microscopy analysis, which demonstrated that ANP resulted in the destruction of mitochondria in the alveolar epithelial cells and induction of auto-phagosome and lysosomes formation (Figure 4), suggesting that NPs might induce autophagy in lung tissue. In this context, multiple immune cells were found to have the capability to locally synthesize ANP and express its corresponding receptors. Notably, ANP has the ability to stimulate macrophages, thus leading to an acceleration of phagocytosis and killing activity via the overproduction of reactive oxygen species (ROS) [27].

The second set of experiments was performed in rats with CHF. The experimental model of CHF, induced by creating an aorto caval fistula, exhibited two distinctive patterns, namely compensated and decompensated HF [1]. Using the isolated liquid-filled-lungs model, we showed that the rate of AFC in compensated CHF was significantly upregulated. In contrast, decompensated CHF rats displayed a dramatically impaired AFC rate. This distinct behavior between the two sub-groups could be attributed to differences in blood pressures, which was lower in decompensated rats as compared to compensated animals [28]. The current study shows that the administration of ANP to rats with compensated CHF attenuated the ability of the lungs to clear edema; conversely, in decompensated CHF, ANP restored this effect (Figure 5). In agreement with this observation, it has been reported that ANP attenuates the severity of both cardiogenic and non-cardiogenic pulmonary edema, presumably by preventing the disruption of the alveolo-capillary barrier and thus attenuating the inflammatory effect [29]. Nevertheless, in rodent lungs, ischemia-reperfusion injury prompted the release of ANP into the circulation with subsequent pulmonary endothelial hyper-permeability and edema formation [30]. The distinct behavior of compensated vs. decompensated CHF in response to ANP could not be attributed to different hypotensive responses of these animals to ANP, as previous studies from our group clearly showed that ANP administration provoked comparable hypotensive responses in both sup-groups of CHF and their sham controls [31]. In rats with compensated CHF, α-ENaC expression in AECII was increased; however, the administration of ANP diminished this positive effect. The same effect was achieved with α1 Na^+^, K^+^-ATPase abundance. Notably, α1 Na^+^, K^+^-ATPase m-RNA was significantly reduced in both groups of compensated CHF. This might be attributed to other factors such as angiotensin that inhibits AFC via c-AMP-mediated downregulation of Na^+^, K^+^-ATPase [32]. Notably, total α1 Na^+^, K^+^-ATPase tended to increase but did not reach statistical significance; nevertheless, we have shown that α1 Na^+^, K^+^-ATPase levels were remarkably increased in the basolateral membranes of AEC in compensated CHF rats [11].

The primary molecular difference between compensated and decompensated CHF lies in the ratio between alpha ENaC levels and the Na^+^, K^+^-ATPase pump. As shown in Figure 7, in AECII isolated from compensated CHF rats, the levels of both alpha ENaC and the Na^+^, K^+^-ATPase pump remained unchanged compared to AECII isolated from sham-operated rats. However, in decompensated rats, alpha ENaC levels increased, while Na^+^, K^+^-ATPase pump levels slightly decreased, creating an imbalance between these key molecules and resulting in impaired alveolar fluid clearance. Additionally, we assessed changes in the immunoreactive levels of phosphodiesterase 5A (PDE5A), an enzyme responsible for the degradation of c-GMP. Our results revealed an intriguing phenomenon: the levels of PDE5A were significantly higher in the compensated group but drastically decreased in the decompensated group, suggesting reduced degradation of c-GMP (see Supplementary Figure S3). Jain et al. have shown that nitric oxide inhibited active sodium transport in AECII via c-GMP-dependent protein kinase [33]. Based on previous knowledge that AP effect is regulated by c-GMP [14], it is reasonable to hypothesize that increased PDE5A levels in compensated CHF accelerated c-GMP degradation with resultant enhancement of AFC. While in decompensated HF, the observed decrease in PDE5A levels inhibited c-GMP degradation, resulting in decreased AFC. Therefore, we hypothesize that the effect of ANP on AFC, which negatively affected AFC and contributed to the formation of pulmonary edema. This observation, along with the proposed hypothesis, warrants further investigation to explore the underlying mechanisms in greater depth.

The set of experiments presented in Figure 8 was designed to determine whether the effect of ANP on αENaC was mediated via ENaC ubiquitination. Our results have shown that in Nedd4-2 co-transfected cells, ANP treatment triggered Nedd4-2 upregulation with consequent αENaC downregulation. In agreement with our observation, Boase et al. demonstrated that knockout of Nedd4-2 in mice resulted in increased ENaC expression and activity in embryonic lung. Therefore, Nedd4-2 is vital for appropriate regulation of ENaC expression [34].

**Figure 9:**
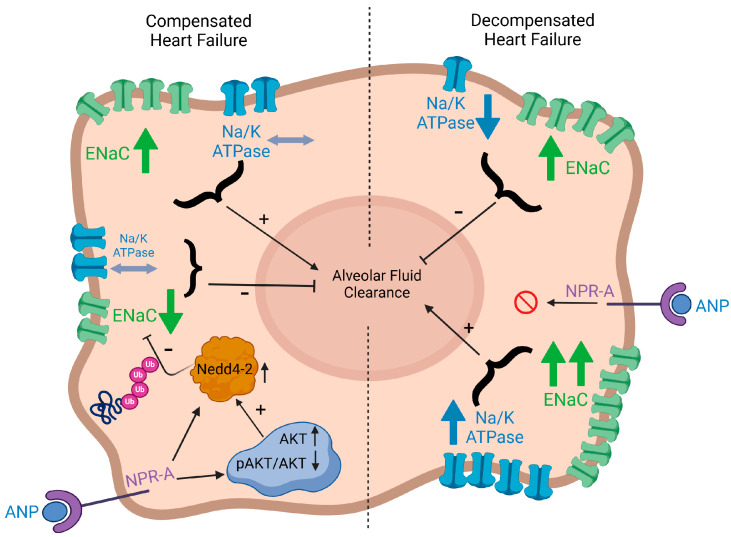
Graphic illustration depicting a model of how natriuretic peptides, αENaC, Na/K-ATPase, Nedd4-2, and AKT in AEC are linked to modulate AFC. AFC: alveolar fluid clearance; AEC: alveolar epithelial cells; ANP: atrial natriuretic peptides; ENaC Ub: ubiquinated ENaC; p-AKT: phosphorylated AKT.

We also demonstrated that ANP upregulated AKT levels in Nedd4-2 co-transfected cells with consequent remarkable decreases in the pAKT/AKT ratio. Therefore, it is conceivable to conclude that ANP plays a role in mediating Nedd4-2 phosphorylation and improving function, resulting in αENaC hyper-ubiquitination along with impaired Na^+^ transport in the lung. Our results are concordant with Lee et al., who showed that overexpression of Nedd4-2 downgraded the activity of ENaC by 50%. Conversely, overexpression of either Akt1 or Sgk1 overcame this inhibitory effect of Nedd4-2 on ENaC [35]. Consistently with our data, it was reported that P-Akt levels were diminished and Nedd4–2 protein expression levels were increased in a model of LPS-injured rat lungs [23]. Since the late 1980s, the treatment of patients with heart failure with reduced ejection fraction (HFrEF) has undergone a major revolution thanks to groups of drugs that have changed the course of the disease, improved the prognosis, and improved the quality of life. These include beta-adrenergic blockers, renin–angiotensin–aldosterone system antagonists (angiotensin-converting enzyme inhibitors (ACEI)/angiotensin receptor blockers (ARB)/angiotensin receptor-neprilysin inhibitor (ARNI), mineralocorticoid receptor antagonists (MRA)), and sodium-glucose transport protein 2 (SGLT2) inhibitors [1]. ARNI, which increases ANP, has been chosen as one of the “Fantastic 4” for the treatment of HFrEF. However, Patients with acute decompensated heart failure (ADHF) were excluded from the evaluation in PARADIGM-HF [36]. The PIONEER-HF included ADHF patients after stabilization, and the study was not powered for clinical outcomes [37]. Notably, the use of recombinant human ANP, carperitide, in acute heart failure remains controversial. To better understand its prognostic impact, large, well-designed clinical trials are needed. As for recombinant BNP, treatment with nesiritide did not reduce 30-day rehospitalization rates or mortality in heart failure patients. Similarly, another recombinant BNP, ularitide, failed to provide clinical benefits, despite employing a different therapeutic approach [38]. Therefore, based on our data showing the positive effect of ANP in decompensated HF rats (Figure 9), it would be of great interest to examine the effects of these therapeutic modalities in decompensated HF rats.

Limitations: Despite its significance, the current study suffers from few limitations: 1—The number of animals in each group is modest; 2—We focused on ANP as it is of more physiological relevance to AFC, yet the impact of BNP on the latter deserves more comprehensive study; 3—The applied model of CHF is high cardiac output; similar studies should be conducted on low cardiac output models such as post myocardial infarction-induced cardiac dysfunction; 4—The downstream mechanisms underlying the impact of ANP on AFC need to be explored more thoroughly.

## 4. Materials and Methods

### 4.1. Animal Care

In this study, we used samples collected from among the same animals utilized in our CHF model, as previously described [15]. Experiments were performed on adult male Sprague–Dawley rats (Harlan Laboratories, Jerusalem, Israel), weighing 300–350 g. Rats were housed in individual metabolic cages in a temperature-controlled room and fed a standard rodent diet and tap water ad libitum. Urinary volume and urinary sodium concentration were measured throughout the entire study period, beginning 4 days prior to surgery. Animals received humane care, in compliance with the guidelines of the Ruth & Bruce Rappaport Faculty of Medicine, Technion, Israel Institute of Technology.

### 4.2. Study Design

**Figure 10:**
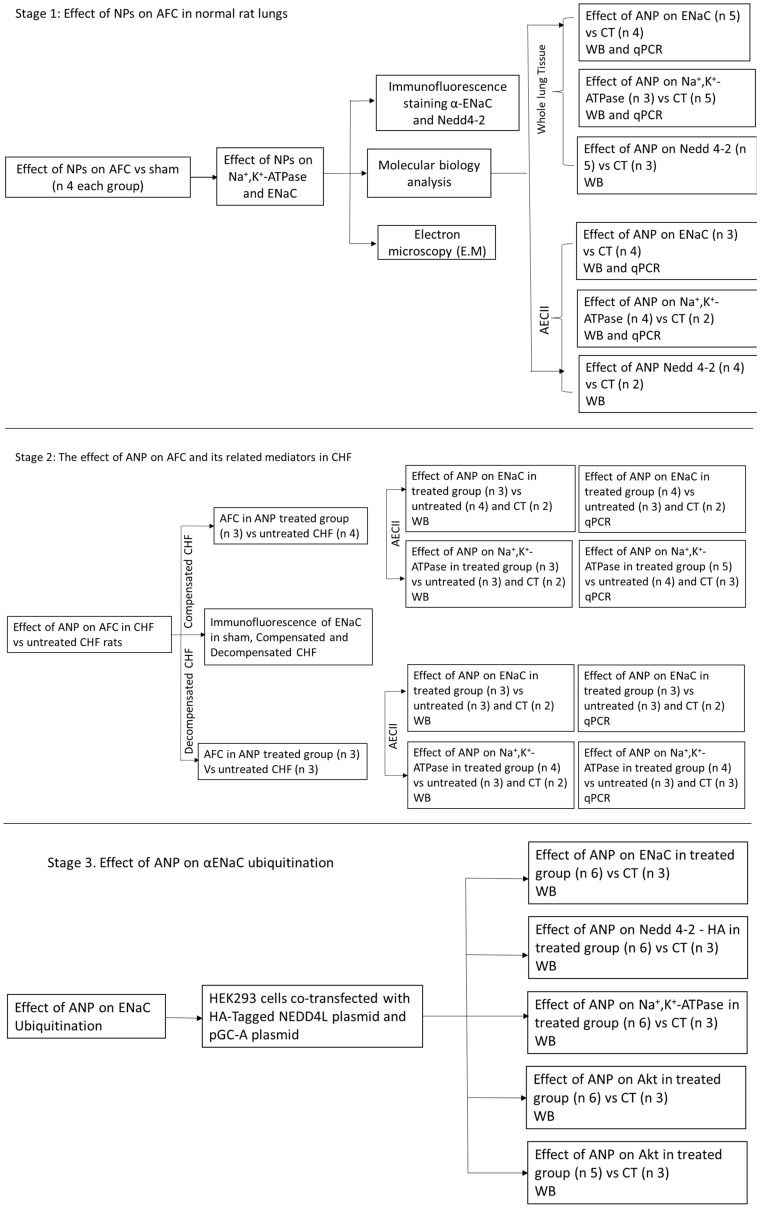
The study design. Flow chart that describes the study design and the three research stages. CT, control; WB, Western blotting; CHF, congestive heart failure; AFC, alveolar fluid clearance; NP, natriuretic peptides.

As depicted in Figure 10, the study is composed of 3 stages. First, to study the effects of NPs on active Na^+^ transport in normal rat lung. Second, to investigate the effect of ANP on AFC and its related mediators in CHF. Third, to explore the effect of ANP on αENaC ubiquitination.

### 4.3. Measurement of Alveolar Fluid Clearance (AFC)

Utilizing the extensively investigated ex vivo model (isolated perfused liquid-filled lung model), AFC was calculated by measuring changes in Evans blue-tagged albumin concentration over time [13,39].

The effects of ANP and BNP on AFC rate were first measured in normal, healthy rat lungs, after which the effects of ANP on AFC were measured in both compensated and decompensated CHF rats. ANP and BNP were administered to the perfusate at a concentration of 10^−4^ M.

### 4.4. Experimental CHF

Heart failure was induced by a surgical creation of a 1.2 mm arteriovenous fistula between the abdominal aorta and inferior vena cava. This procedure is routinely used in our laboratory to induce both compensated and decompensated heart failure [13,28]. Following surgery, rats were allowed to recover and placed in metabolic cages for 1 week, after which those with aorto caval fistula were divided into two subgroups according to their daily absolute rate of sodium excretion (UNaV): (1) rats with decompensated CHF (UnaV < 200 µEq/24 h) and (2) rats with compensated CHF (UNaV > 1200 µEq/24 h) [40].

### 4.5. Cell Isolation, Culture, and Treatment

Alveolar epithelial type II (AECII) cells were isolated from normal ANP-treated rats and their controls that were treated with saline, as well as from the compensated and decompensated CHF rats that were treated with ANP and their controls. AECII cells were isolated according to the method of Dobbs et al. [40], with minor modifications [35,41,42]. Briefly, cells were isolated by elastase digestion of lung tissue and then differentially adhered onto IgG-coated plates. AECII cells were seeded onto plastic culture dishes and cultured in a 5% CO_2_, 95% air atmosphere in DMEM containing 10% fetal bovine serum penicillin (100 U/mL), streptomycin (0.1 mg/mL), gentamycin (0.05 mg/mL), amphotericin B (0.25 µg/mL), and L-Glutamine (2 mM). The plating density of cells varied depending on the purpose of the experiment. Cell pellets were collected after 24 h. The markers used to characterize AECII were depicted in Figure S1.

### 4.6. Lung Fixation and Histology

The lungs of sham non-CHF rats that were treated with ANP and their controls were fixed via carotid artery perfusion, first with 40 mL phosphate-buffered saline (0.02 M PBS, pH 7.4) containing heparin (5 U/mL) and then with 220 mL of ice-cold 4% paraformaldehyde in 0.1 M PBS containing 4% sucrose at pH 7.5. Fixation solution was also instilled into the alveolar spaces via tracheotomy. Lungs from the different experimental groups were removed and embedded in 10% neutral-buffered formalin. Lung tissue samples were then progressively dehydrated in graduated alcohol concentrations (70–100%), embedded in paraffin, and stained with hematoxylin and eosin (H&E) for microscope observation.

### 4.7. Immunofluorescence Staining

Five-micrometer-thick paraffin sections of the lung tissues were deparaffinized, rehydrated, and placed on slides that were subjected to antigen retrieval by using Proteinase K (ab64220, Abcam; Cambridge, UK) for 5 min. Slides were then incubated with 5% normal donkey serum (NDS) in phosphate-buffered saline (PBS) containing 0.3% Tween-20 for 60 min to block non-specific binding and then incubated overnight at 4 °C with primary antibodies diluted in blocking solution and directed against anti-αENac (1:100, rabbit, Invitrogen, PA1-920A, Carlsbad, CA, USA), and anti-Nedd4-2 (1/40, #ab46521, Abcam). CyTM3 Donkey Anti-Rabbit IgG was used as the secondary antibody (Jackson ImmunoResearch, Philadelphia, PA, USA) together with DAPI Fluoromount-G^®^ for nuclear staining. Images were captured using a Widefield Zeiss Upright microscope and analyzed with Zen software (ZEN 2.3 SP1). Representative images of lung tissue were obtained at ×20 magnification.

### 4.8. Immunofluorescence of Isolated AECII

AECII isolated from sham, compensated, and decompensated CHF were plated on coverslips for 24 h. Subsequently, the cells were rinsed with ice-cold PBS and fixed with 4% PFA for 15 min at 25 °C. Cells were blocked with 1% bovine serum albumin 0.3% and Tween 20 for 1 h at room temperature, incubated with anti-αENaC (1:100, rabbit, Invitrogen, PA1-920A) at 4 °C overnight, and then incubated with CY3-conjugated secondary antibody (Jackson Laboratories, Sacramento, CA, USA) for 1 h at room temperature. Nuclei were stained with DAPI (Invitrogen, Thermo Fisher Scientific, Waltham, MA, USA). Finally, the fluorescence was visualized using a Zeiss LSM 880 confocal microscope (Carl Zeiss Microscopy GmbH, Jena, Germany) and analyzed with Zen software. Representative images of AECII were obtained at ×20 magnification.

### 4.9. Western Blot Analysis

Pulmonary tissue samples from the normal ANP-treated group and their control were homogenized on ice. The homogenized tissue and AECII pellets collected from the various experimental groups were then lysed in RIPA buffer (150 mM NaCl, 1% NP40, 50 mM Tris pH 8.0, 0.5% sodium deoxycholate, and 0.1% SDS) supplemented with a cocktail of protease inhibitors (Roche, #11836170001, Basel, Switzerland) in rotation at 4 °C for 20 min and then centrifuged at 4 °C for 15 min at 12,000 RPM. The cleared supernatant was collected, and the protein concentration was determined using Bradford reagent (Sigma-Aldrich Israel Ltd., Rehovot, Israel). Equal amounts of extracted proteins (30–50 μg) were loaded and run by electrophoresis on a 10% SDS–polyacrylamide gel and then transferred to a nitrocellulose membrane. The membranes were incubated in blocking buffer, TBS-T (Tris-buffered saline, 0.1% Tween 20) containing 5% (*w*/*v*) BSA and probed with the appropriate primary antibodies: anti-αENac (1:500, rabbit, Invitrogen, PA1-920A), anti-αNa^+^, K^+^-ATPase (1:1000, mouse, ab7671, Abcam), anti-Nedd4-2 (1/500, #ab46521, Abcam), anti-Phospho-Akt (1:1000, rabbit, 9271, Cell Signaling, Danvers, MA, USA), anti-AKT (1:1000, rabbit, 9272, Cell Signaling), anti-HA Tag (1:1000, rabbit, 3724, Cell Signaling), and anti-GAPDH (1:500, mouse, sc-32233, Santa Cruz Biotechnilogy, Dallas, TX, USA) diluted in blocking solution. After washing with TBS-T, the immunoreactive proteins were visualized with horseradish-conjugated goat anti-rabbit (1:25,000, 111-035-144, Jackson) and donkey anti-mouse (1:10,000, 715-035-151, Jackson) IgG secondary antibodies and chemiluminescent substrate.

### 4.10. RNA Extraction and Real-Time qPCR

Total RNA was isolated from the AECII cell pellet using TRIzol^®^ Reagent (Life Technologies; Carlsbad, CA, USA) according to the manufacturer’s instructions and quantified by spectrophotometry using NanoDrop2000. After oligo (dT)–primed reverse transcription of 1000 ng total RNA, the resulting single-stranded cDNA was used for PCR. PCR conditions were as follows: an initial denaturation step at 95 °C for 3 min, 30 cycles of denaturation at 95 °C for 30 s, and hybridization at 60 °C for 30 s followed by elongation at 72 °C for 1 min. Finally, the PCR reaction was terminated by incubation at 72 °C for 5 min. GAPDH protein was used as an internal standard. The following primers were used as shown in Table 1.

### 4.11. Electron Microscopy

We used a transmission electron microscope (TEM) to detect ultrastructural changes in AECII after ANP treatment. Normal ANP-treated rats and their controls were anesthetized and perfused with a solution composed of 2.5% glutaraldehyde in 0.1 mol/L phosphate buffer (pH 7.4). Thereafter, lung tissues were harvested and diced. Three or four pieces of approximately one mm^3^ each were immersed in the perfusion solution for 2 h for fixation and then soaked overnight in a solution without glutaraldehyde. The specimens were dehydrated through a graded ethanol series. Each specimen was embedded in epoxy resin, after which ultrathin sections (90 nm) were stained with uranyl acetate and lead citrate before examination using TEM.

### 4.12. Cell Culture, Plasmids, and Transfections

Full-length human pGC-A (amino acids 33–1061 of NCBI Reference Sequence NP_000897.3) was amplified using pFastBac1-pGC-A plasmid (Addgene, 186626, Watertown, MA, USA) as a template, using Q5 high-fidelity polymerase (NEB-M0492) (forward: 5’caagcttggtaccgagctcggatccatgaaaactattatcgccctgtcttac 3’. Reverse: 5’ acactatagaatagggccctctagattatccacgggtgctgctac 3’). The gene encoding the pGC-A protein was cloned into the pcDNA3.1 vector. The pcDNA3.1 vector was also amplified by Q5 high fidelity polymerase using the following primers: forward: 5’ ccgtggataatctagagggccctattctatagtg 3’ and reverse: 5’ tagttttcatggatccgagctcggtacc 3’. Both products of the insert and the PcDNA3.1 vector were assembled using NEBuilder HiFi DNA Assembly (E2621). The final vector was sequence-verified by GenScript.

To determine the effects of ANP on αENaC ubiquitination in human embryonic kidney, 293 (HEK293) cells were co-transfected with HA-tagged NEDD4L plasmid (Addgene, #2700) and pGC-A plasmid, both cloned in our laboratory. Cells were cultured in 12-well plates, whereby each well was transfected with 0.5 μg DNA (0.25 μg/plasmid) using 1 mg/mL polyethylenimine (5:1 ratio of PEI:DNA). At 24 h post transfection, cells were treated with 100 uM ANP. After 24 h, cell pellets were collected, and changes in αENaC and Nedd4-2 levels were detected using Western blot analysis. Cell pellets were lysed in RIPA buffer supplemented with a cocktail of protease inhibitors, samples were prepared as discussed previously for Western blot analysis, and the resulting membranes were incubated with the following primary antibodies: anti-αENaC, anti-HA Tag, anti-Phospho-Akt, anti-AKT, anti-αNa^+^, and K^+^-ATPase.

### 4.13. Statistical Analysis

All graphs, calculations, and statistical analyses were conducted using GraphPad Prism software, version 8.0 (GraphPad Software, San Diego, CA, USA). Data are shown as mean ± standard error of the mean (SEM). One-way analysis of variance (ANOVA) and the Kruskal–Wallis test for more than two groups, adjusted for multiple comparisons using the Dunn’s post hoc test, were applied. The nonparametric Wilcoxon–Mann–Whitney test was applied to compare the effects of ANP in compensated and decompensated CHF rats. Comparisons between two parametric groups were performed using an unpaired Student’s *t*-test after testing for equality of variances. A *p* value < 0.05 was considered statistically significant.

## 5. Conclusions

In conclusion, we provide evidence that NPs compromised AFC by downregulating active Na^+^ transport in the alveolar epithelium. This effect took place by enhancing the ubiquitination and degradation of α-ENaC in the lungs through upregulation of Nedd4-2 via the AKT/SGK1 pathway.

Furthermore, we demonstrated that in compensated CHF, ANP impaired the AFC by enhancing ENaC ubiquitination at the apical membrane. In contrast, in decompensated CHF, ANP restored AFC by upregulating components of the alveolar active sodium transport pathway, thereby alleviating lung edema development.

It is reasonable that in ANP-treated decompensated CHF, Nedd4-2 function may be diminished, leading to reduced ENaC ubiquitination. This effect likely results from increased phosphorylation of both AKT and SGK1 kinases.

Future research is needed to explore alterations in the AKT/SGK1–Nedd4-2 pathway following ANP treatment. Furthermore, investigating potential variations in ANP/BNP receptor expression could help explain the differing ANP responses between compensated and decompensated CHF.

## Figures and Tables

**Table 1:** The primers used for RT-PCR.

αENaC	F (5’-TCCTGCAACCAGGCGAATTA-3’) R (5’-TCAGGGACAGACCGTTGTTG-3’)
αNa^+^, K^+^-ATPase	F (5’-TGCTCCGACAAGACTGGAAC-3’) R (5’-GTCAAAGGAGACCCCACTCTG-3’)
GAPDH	F (5′-GTGCCAGCCTCGTCTCATAG-3′) R (5′-GAGAAGGCAGCCCTGGTAAC-3′)

## Data Availability

Data are contained within the article.

## Supplementary Materials

The following supporting information can be downloaded at: https://www.mdpi.com/article/10.3390/ijms26073374/s1.

## Abbreviations

AEC	Alveolar Epithelial Cells
AFC	Alveolar Fluid Clearance
AKT	Protein kinase B
ARNI	Angiotensin Receptor-Neprilysin Inhibitor
ANP	Atrial Natriuretic Peptide
ATP	Adenosine Triphosphate
BNP	Brain Natriuretic Peptide
BSA	Bovine Serum Albumin
c-GMP	Cyclic guanosine monophosphate
CHF	Congestive Heart Failure
CNP	C-type Natriuretic Peptide
CPE	Cardiogenic Pulmonary Edema
DMEM	Dulbecco’s Modified Eagle Medium
ENaC	Epithelial Na+ Channel
HA-tagged	Human Influenza HemAgglutinin
HF	Heart Failure
Na^+^, K^+^-ATPase	Na, K-ATPase Sodium Potassium Pump
Nedd 4-2	Neural precursor cell expressed developmentally down-regulated protein 4
NPs	Natriuretic Peptides
SDS	Sodium Dodecyl Sulfate
SGK1	Serum and Glucocorticoid-regulated Kinase
TBS	Tris-Buffered Saline
