# Supplementary material for: The Effects of the Natriuretic Peptide System on Alveolar Epithelium in Heart Failure
Source: Int J Mol Sci. 2025 Apr 4;26(7):3374. doi: 10.3390/ijms26073374 (PMC11989889; doi:10.3390/ijms26073374)
Supplement: Supplementary file 1 [file ijms-26-03374-s001.zip › ijms-3485481-supplementary.pdf]

Figure 1S

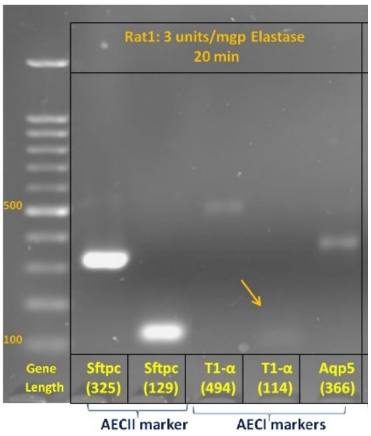

Figure 1S: AECII is characterized by the presence of the SftPC gene, while AECI expresses only T1-α and Aqp5.

SftPC: Surfactant Protein C, a specific AECII marker gene. T1-α: a specific AECI marker gene. AECII: Alveolar Epithelial Cells.

Primers that were used

SFTPC primers:

Forword TATGACTACCAGCGGCTCCT . Reverse CTTTGCGGAGGGTCTTTCCT

T1-α primers :

Forword: CCATCGGTGCGCTAGAAGAT . Reverse GGCAAGGTGGAAGCTCTCTT

Aqp5 Primers:

Forward: GGCCACATCAATCCAGCCATTA . Revers GGCTGGGTTCATGGAACAGCC

Figure 2S

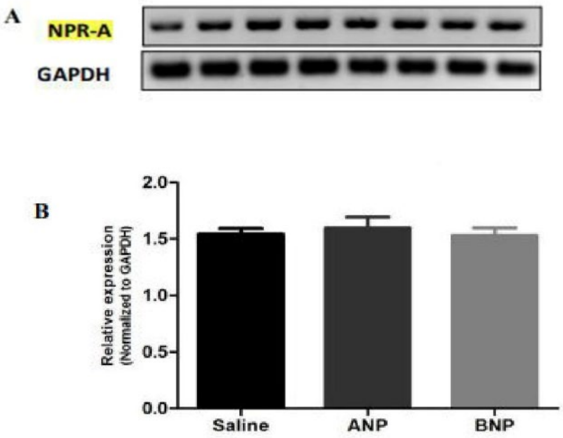

Figure 2S: The expression of NPR-A in lung tissue of ANP, BNP treated rats and control group. (A) mRNA expression of NPR-A. GAPDH expression was used for normalization.

Quantification of PCR analysis for NPRA mRNAs is depicted in (B). There were no statistical differences in NPR-A mRNA levels between ANP/BNP treated group and their controls. Results are expressed as mean ± SEM.
NPRA: Natriuretic Peptide Receptor-A. ANP: Atrial Natriuretic Peptide. Brain Natriuretic Peptide.

Figure 3S

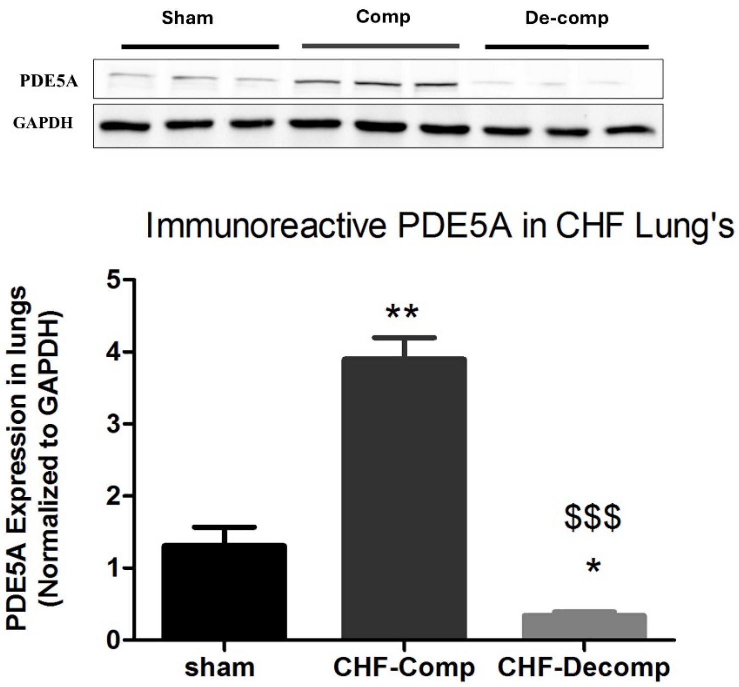

Figure 3S: Expression of PDE5A in lung tissue of compensated, decompensated CHF and sham controls. (A) Representative abundance (western blot) of PDE5A in the lung tissues of compensated and decompensated CHF rats and their sham controls. (B) Quantification of PDE5-A abundance. Results are expressed as mean ± SEM. N-3 in each study group.
(*) represents a significant difference between decompensated CHF group and sham controls, P=0.0206;
(**) represents a significant difference between compensated CHF group and sham controls P= 0.028.
($$$) represents significant differences of decompensated CHF group vs. compensated CHF group P=0.0003.
